# Liuwei Dihuang Pills Enhance the Effect of Western Medicine in Treating Diabetic Nephropathy: A Meta-Analysis of Randomized Controlled Trials

**DOI:** 10.1155/2016/1509063

**Published:** 2016-02-21

**Authors:** Lan Lin, Qiuhong Wang, Yongxin Yi, Shihan Wang, Zonglin Qiu

**Affiliations:** ^1^Department of Endocrinology, Guang'anmen Hospital, China Academy of Chinese Medical Sciences, No. 5 Beixiange, Xicheng District, Beijing 100053, China; ^2^Department of Cardiology, Guang'anmen Hospital, China Academy of Chinese Medical Sciences, Beijing 100053, China

## Abstract

*Objectives.* To assess the effectiveness and adverse effects of adding Liuwei Dihuang Pills (LDP) to Western medicine for treating diabetic nephropathy.* Methods.* Studies were retrieved from seven electronic databases, including PubMed, Embase, The Cochrane Library, CBM, CNKI, Chinese Scientific Journal Database (VIP), and Wanfang Data until November 2015. Study selection, data extraction, quality assessment, and data analyses were conducted according to Cochrane standards. Meta-analysis was performed on the overall therapeutic efficacy of hyperglycemia and renal functions, and the study also analyzed adverse events.* Results.* A total of 1,275 patients from 18 studies were included. The methodological quality of these included trials was generally low. We found that adding LDP can lower patients' FBG (MD: −0.36 [−0.46, −0.25], *P* < 0.00001), PBG (MD: −1.10 [−1.35, −0.85], *P* < 0.00001), and HbA1c (MD: −0.14 [−0.49, 0.21], *P* = 0.43). There were also improvements in lowering patients' BUN (MD: −0.67 [−0.89, −0.45], *P* < 0.00001), SCr (MD: −0.96 [−1.53, −0.39], *P* < 0.00001), 24 h UTP (SMD: −1.26 [−2.38, −0.15], *P* < 0.00001), UAER (MD: −26.18 [−27.51, −24.85], *P* < 0.00001), and UmAlb (SMD: −1.72 [−2.67, −0.77], *P* < 0.00001).* Conclusion.* There is encouraging evidence that adding LDP to Western medicine might improve treatment outcomes of diabetic nephropathy, including hyperglycemia and renal functions. However, the evidence remains weak. More rigorous high-quality trials are warranted to substantiate or refute the results.

## 1. Introduction

Diabetic nephropathy (DN) is a widely recognized microvascular complication of diabetes and almost the leading cause of end-stage kidney failure worldwide responsible for morbidity and mortality [1]. If the damage to the kidney and proteinuria is irreversible, it will evolve into End-Stage Renal Disease. However, exact pathogenesis of DN is still unclear and it is difficult for us to cure DN. At present diet management, control of blood pressure and blood sugar, and blood fat treatment are the foundation treatment for DN. Furthermore, an adequate control of high blood pressure and treatment of microalbuminuria are the major therapeutic targets [2]. To achieve adequate blood pressure control, a combination therapy with different classes of antihypertensive agents is often necessary, especially including angiotensin-converting enzyme inhibitors (ACEIs) and angiotensin receptor blockers (ARBs) [3]. ACEIs and ARBs have been demonstrated to protect renal function of DN but are not enough to delay or retard the progression of DN; therefore, exploring feasible drugs is the hotspot of medical research at present.

Currently, with increasing application of complementary and alternative medicine (CAM) worldwide, traditional Chinese medicine (TCM) has become more popular and has drawn more attention [4–7]. TCM has lots of advantages over the conventional medical approaches in the prevention of diabetic complications because of less toxicity and/or side effects [8–10]. TCM is becoming increasingly popular and widely used among patients with DN [11].

Liuwei Dihuang Pills (LDP), a traditional Chinese herbal formula containing six commonly used herbs (*Rehmannia glutinosa*,* Cornus officinalis* Sieb., Common Yam Rhizome,* Alisma orientalis*, Tree Peony Bark, and* Poria cocos*), are widely used to DN-related symptoms in clinical practice for centuries in China. Three of the six ingredients in the formula are nutrients, while the other three facilitate drainage, enrich yin, nourish the kidney (Shen), and thereby address the root cause of diabetes according to the Chinese medicine theory [12]. Currently, LDP combined with Western medicine has been widely used as an alternative and effective method to treat or prevent diabetic nephropathy in China. Up to now, lots of studies have been published about the effects of LDP combined with conventional drugs for diabetic nephropathy. It is necessary for us to compare the effect of such combinations to the use of Western medicine alone. This report aims to evaluate the beneficial and adverse effects of LDP combined with conventional drugs for the treatment of DN in randomized trials.

## 2. Materials and Methods

### 2.1. Database and Search Strategies

Initial searches were performed by 2 authors independently. We selected all the clinical trials about LDP used for treating DN in the Chinese National Knowledge Infrastructure (CNKI), Chinese Scientific Journal Database (VIP), Wanfang Data, Chinese Biomedical Literature Database (CBM), PubMed, Embase, and the Cochrane Central Register of Controlled Trials in The Cochrane Library. All of those searches ended on November 30, 2015. Search terms used were diabetes mellitus, DN, and chronic kidney impairment, combined with LDP. Additionally, we checked bibliographies of retrieved articles and prior reviews on the subject for additional references. We contacted the authors of included trials for missing information when necessary.

### 2.2. Inclusion and Exclusion Criteria

All the randomized controlled trials (RCTs) based on LDP combined with Western drugs or Western conventional therapeutics compared with corresponding Western drugs or Western conventional therapeutics in patients with DN were included. No restrictions on language, population characteristics, or publication type were enforced. The primary outcome measure that the current study examined was the overall clinical efficacy of LDP for hyperglycemia and impairments of renal functions. A secondary outcome that was assessed was frequency of adverse events. Articles that duplicated the same groups of participants in another publication were excluded.

### 2.3. Data Extraction and Quality Assessment

Two authors conducted the literature searching (Q. H. Wang and Y. X. Yi), two conducted study selection (Q. H. Wang and S. H. Wang), and two conducted data extraction (Y. X. Yi and Z. L. Qiu) independently. The extracted data included authors, title of the study, year of publication, patients' characteristics and the studies' designs, sample sizes, details of intervention, details of control interventions, outcomes, adverse effects for each study, and intervention durations. Discrepancies were resolved by discussion and consensus was reached through a third party (L. Lin). We assessed the methodological quality of trials using the criteria of the Cochrane Handbook for Systematic Review of Interventions, version 5.2.3 (Q. H. Wang and S. H. Wang) [13]. The items included the following 6 aspects: random sequence generation (selection bias), allocation concealment (selection bias), blinding of participants and personnel (performance bias), blinding of outcome assessment (detection bias), incomplete outcome data (attrition bias), and selective reporting (reporting bias).

### 2.4. Data Synthesis

All data analyses were conducted using the Review Manager 5.2 software provided by the Cochrane Collaboration. The outcomes were analyzed as continuous variables using fixed- or random-effect models, and the results for overall clinical efficacy were reported as weighted mean difference (WMD), 95% confidence intervals (95% CIs), and pooled odds ratio (OR). Meta-analysis was performed if the intervention, control, and outcome were the same or similar. It was considered to be indicators of a substantial level of heterogeneity when *I* ^2^ > 50% or *P* < 0.1. In the absence of significant heterogeneity, data were pooled using a fixed-effect model (*I* ^2^ > 50%), and, otherwise, data were pooled using a random-effect model (50% < *I* ^2^ > 50%[14]. The relative strength of treatment efficacy was illustrated by forest plots.

## 3. Results

### 3.1. Description of Included Trials

**Figure 1:**
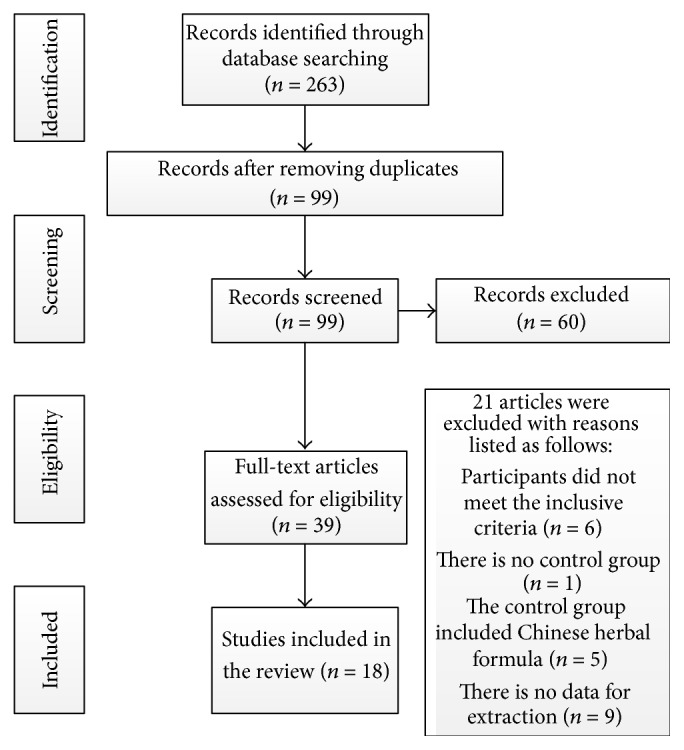
Study selection process.

99 trials were screened out from electronic and manual searches in the seven databases. The screening process is summarized in a flow diagram (Figure 1). Only 18 RCTs [15–32] were included. All of the 18 trials were conducted in China and published in Chinese between 2004 and 2015. The characteristics of the 18 included trials were summarized in Table 1.

The 18 RCTs involved 1257 patients with diabetic nephropathy. 14 trials [15–28] specified two diagnostic criteria of diabetic nephropathy, one trial [15] used 1985 WHO criteria for the diagnosis of diabetes mellitus, 2 trials [16, 17] used Chinese Guidelines for the Prevention of Diabetes Mellitus-2007, 11 trials [18–28] used 1999 WHO criteria for the diagnosis of diabetes mellitus and 1987 Mogensen Criteria for the diagnosis of diabetic nephropathy, and the other four trials [29–32] only demonstrated patients with diabetic nephropathy without detailed information.

The interventions included LDP combined with antihypertensive or routine treatment drugs. The controls included three types of groups, including antihypertensive drugs, routine treatment, and antihypertensive drugs combined with routine treatment. The range of participants' mean ages in the various RCTs was from 39.9 to 66.4 years. The total treatment duration was in the range of 21 to 360 days. The outcome measures included Scr, BUN, UmAlb, UAER, 24 h urine protein, FBG, PBG, and HbA1c. Adverse effect was described in detail. The main finding of the trials showed that the use of LDP plus Western drugs had beneficial effects for the prevention and therapy of diabetic nephropathy.

### 3.2. Methodological Quality of Induced Trials

The quality assessments are summarized in Table 2. The randomized allocation of participants was mentioned in all RCTs. However, only 6 trials [16, 22, 24, 28, 30, 32] stated the methods for sequence generation by using random number tables or coin tossing or drawing lots. Two trials [17, 23] described inappropriate methods of randomization by order of hospital registration. The others did not mention the sequence generation processes adequately. Thus we failed to judge whether it was conducted properly or not because of insufficient information. No trial stated the double-blind principle and allocation concealment. Only one trial [23] mentioned single blinding but gave no details of either participants' or investigators' or assessors' blinding. Only one trial [16] reported dropouts or withdrawals. Selective reporting was generally unclear in the RCTs due to lacking data for the current research team. All the trials did not mention follow-up. The pretrial estimation of sample size was not mentioned in all trials. We tried to contact the authors for further information but regrettably we have got no information.

### 3.3. Effect of Interventions

#### 3.3.1. Improvement of Hyperglycemia

We analyzed the RCTs that studied the benefits of LDP for FPG, PBG, and HbA1c.

*(1) Improvement of FPG*. Six studies [17, 18, 22, 23, 28, 29] including 371 participants used the levels of FPG as an outcome measure. These trials showed homogeneity in the consistency of the trial results (chi-square = 7.00; *P* = 0.22; *I* ^2^ = 29%). Therefore, a fixed-effect model should have been used for statistical analysis. A meta-analysis showed a significant difference for the LDP combined with conventional therapies group (MD: −0.36 [−0.46, −0.25]; *P* < 0.00001), which demonstrated that LDP combined with conventional therapies group was superior to the conventional therapies taken by the control groups (Table 3).

*(2) Improvement of PBG*. Three studies [17, 18, 28] including 194 participants used the levels of PBG to measure the outcome. The trials showed homogeneity in the consistency of the trial results (chi-square = 0.79; *P* = 0.67; *I* ^2^ = 0%). Therefore, a fixed-effect model should have been used for statistical analysis. A meta-analysis showed a significant beneficial effect of LDP combined with conventional therapies compared with conventional therapies in decreasing the level of PBG (MD: −1.10 [−1.35, −0.85]; *P* < 0.00001) (Table 4).

*(3) Improvement of HbA1c*. Two studies [17, 22] including 112 participants used the levels of HbA1c to measure the outcome. The trials showed homogeneity in the consistency of the trial results (chi-square = 0.07; *P* = 0.79; *I* ^2^ = 0%). Therefore, a fixed-effect model should have been used for statistical analysis. A meta-analysis showed that HbA1c measurements also showed greater efficacy for LDP combined with conventional therapies group (−0.14 [−0.49, 0.21]; *P* = 0.43) (Table 5).

#### 3.3.2. Improvement of Renal Functions

We analyzed the RCTs that measured blood urea nitrogen, serum creatinine, UAER, 24 h urine protein quantitation, and UmAlb.

*(1) Improvement of BUN*. Four studies [21, 23, 24, 31] including 376 participants used the levels of BUN to measure the outcome. The trials showed heterogeneity in the consistency of the trial results (chi-square = 10.94; *P* = 0.01; *I* ^2^ = 73%). Therefore, a random-effect model should have been used for statistical analysis. A meta-analysis showed a significant beneficial effect of LDP combined with conventional therapies compared with conventional therapies in decreasing the level of BUN (MD: −0.67 [−0.89, −0.45]; *P* < 0.00001) (Table 6).

*(2) Improvement of Scr*. Six studies [21, 23, 24, 27, 30, 31] including 458 participants used the levels of Scr to measure the outcome. Four studies including 210 participants [21, 23, 27, 30] compared the combination of LDP treatment plus routine treatment drugs with routine treatment drugs. The trials showed homogeneity in the consistency of the trial results (chi-square = 3.40; *P* = 0.33; *I* ^2^ = 12%). Therefore, a fixed-effect model should have been used for statistical analysis. A meta-analysis showed a significant beneficial effect of LDP plus routine treatment drugs compared with routine treatment drugs in decreasing the level of Scr (MD: −0.60 [−0.89, −0.30]; *P* < 0.0001) (Table 7). Two studies [24, 31] including 248 participants compared the combination of LDP treatment plus Losartan Potassium with Losartan Potassium. The trials showed heterogeneity in the consistency of the trial results (chi-square = 22.72; *P* < 0.00001; *I* ^2^ = 96%). Therefore, a random-effect model should have been used for statistical analysis. A meta-analysis showed a significant beneficial effect of LDP combined with Losartan Potassium compared with Losartan Potassium in decreasing the level of Scr (MD: −1.14 [−2.45, 0.18]; *P* = 0.09) (Table 7).

*(3) Improvement of 24 h UTP*. Five studies [18, 20, 25, 27, 32] including 327 participants used the levels of 24 h UTP to measure the outcome. Four studies [18, 20, 25, 27] including 207 participants compared the combination of LDP treatment plus conventional therapies with conventional therapies. The trials showed homogeneity in the consistency of the trial results (chi-square = 1.20; *P* = 0.75; *I* ^2^ = 0%). Therefore, a fixed-effect model should have been used for statistical analysis. A meta-analysis showed a significant beneficial effect of LDP plus conventional therapies compared with conventional therapies in decreasing the level of 24 h UTP (SMD: −0.67 [−0.95, −0.38]; *P* < 0.00001) (Table 8). One study [32] including 120 participants compared the combination of LDP plus telmisartan plus routine treatment with telmisartan plus routine treatment. The homogeneity in the consistency of the trial results is not applicable (*Z* = 11.90; *P* < 0.00001). A meta-analysis showed a significant beneficial effect of LDP treatment plus telmisartan plus routine treatment compared with telmisartan plus routine treatment in decreasing the levels of 24 h UTP (SMD: −3.48 [−4.05, −2.90]; *P* < 0.00001) (Table 8).

*(4) Improvement of UmAlb*. Five studies [16, 19, 22, 24, 29] including 321 participants used the levels of UmAlb to measure the outcome. Three studies including 202 participants [16, 24, 29] compared the combination of LDP treatment plus conventional therapies with conventional therapies. The trials showed homogeneity in the consistency of the trial results (chi-square = 0.66; *P* = 0.72; *I* ^2^ = 0%). Therefore, a fixed-effect model should have been used for statistical analysis. A meta-analysis showed a significant beneficial effect of LDP plus conventional therapies compared with conventional therapies in decreasing the level of UmAlb (SMD: −1.37 [−1.68, −1.06]; *P* < 0.00001) (Table 9). One study [19] including 44 participants compared the combination of LDP treatment plus Perindopril plus routine treatment with Perindopril plus routine treatment. The homogeneity in the consistency of the trial results is not applicable (*Z* = 7.76; *P* < 0.00001). A meta-analysis showed a significant beneficial effect of LDP treatment plus Perindopril plus routine treatment compared with Perindopril plus routine treatment in decreasing the levels of UmAlb (SMD: −4.70 [−5.89, −3.52]; *P* < 0.00001). Another study [22] including 75 participants compared the combination of LDP plus enalapril with enalapril. The homogeneity in the consistency of the trial results is not applicable (*Z* = 1.17; *P* = 0.24). A meta-analysis showed a significant beneficial effect of LDP plus enalapril compared with enalapril in decreasing the levels of UmAlb (SMD: −0.27 [−0.73, 0.18]; *P* = 0.24) (Table 9).

*(5) Improvement of UAER*. Eight studies [15, 16, 23, 24, 26–28, 31] including 625 participants used the levels of UAER to measure the outcome. Four studies [23, 26–28] including 246 participants compared the combination of LDP treatment plus routine treatment drugs with routine treatment drugs. The trials showed homogeneity in the consistency of the trial results (chi-square = 13.04; *P* = 0.005; *I* ^2^ = 77%). Therefore, a random-effect model should have been used for statistical analysis. A meta-analysis showed a significant beneficial effect of LDP combined with routine treatment compared with routine treatment in decreasing the level of UAER (MD: −43.65 [−45.73, −41.58]; *P* < 0.00001) (Table 10). Two studies [24, 31] including 248 participants compared the combination of LDP treatment plus Losartan Potassium with Losartan Potassium. The trials showed homogeneity in the consistency of the trial results (chi-square = 0.60; *P* = 0.44; *I* ^2^ = 0%). Therefore, a random-effect model should have been used for statistical analysis. A meta-analysis showed a significant beneficial effect of LDP combined with Losartan Potassium compared with Losartan Potassium in decreasing the level of UAER (MD: −27.30 [−30.01, −24.60]; *P* < 0.00001) (Table 10). Two studies [15, 16] including 131 participants compared the combination of LDP treatment plus antihypertensive drugs plus routine treatment with antihypertensive drugs plus routine treatment. The trials showed heterogeneity in the consistency of the trial results (chi-square = 31.42; *P* < 0.00001; *I* ^2^ = 97%). Therefore, a random-effect model should have been used for statistical analysis. A meta-analysis showed a significant beneficial effect of LDP treatment plus antihypertensive drugs plus routine treatment compared with routine treatment with antihypertensive drugs in decreasing the level of UAER (MD: −4.61 [−6.88, −2.34]; *P* < 0.0001) (Table 10).

### 3.4. Publication Bias

The number of trials was too small to conduct any sufficient additional analysis of publication bias.

### 3.5. Adverse Effects

In total, five of the eighteen trials mentioned the presence or absence of adverse effects [18, 22, 24, 31, 32]. Two trials reported the incidence of adverse events, whereas 3 trials reported that no adverse events occurred in the treatment groups compared with the control groups. One trial [22] reported slight cough (*n* = 1) in the treatment group. Another trial [18] indicated that the treatment group presented with hypoglycemia (*n* = 1) and gastrointestinal tract reaction (*n* = 1) and that the control group presented with hypoglycemia (*n* = 2) and gastrointestinal tract reaction (*n* = 2). In general, adverse events were rare and minor.

## 4. Discussion

There is no specific treatment of diabetic nephropathy. Currently, comprehensive treatments including diet management, control of blood pressure, control of blood sugar, and lipid adjusting treatment have been commonly used. Some corresponding measures taken to intervene actively against some risk factors may reduce proteinuria and delay the occurrence of albuminuria. It is considered that active treatment can prevent or delay the progress of diabetic nephropathy at any stage, especially in the early period. ACEI and ARB drugs are widely used in the treatment of DN, but they are not enough to delay or retard the progression of DN. TCM has lots of advantages over the conventional medical approaches in the prevention and treatment of DN [9, 10].

Based on the meta-analyses of the outcome, LDP may have positive effects for lowering FBG, PBG, and HbA1c and improving renal function. LDP as an adjunctive treatment to conventional drugs significantly improved blood sugar and renal function in patients with DN. However, due to the low-quality methodology and potential publication bias, we cannot draw a definite conclusion of the beneficial effectiveness of LDP combined with Western drugs in preventing and treating diabetic nephropathy. Our positive findings should be interpreted conservatively.

Firstly, in terms of the current evaluative standards, all of the included studies were of low methodological quality. Bias may exist in many areas, such as an unclear method of random sequence, no mention of allocation concealment, and being double-blind. It is also possible that bias was produced by the link of distribution, implementation and measurement, statistics, and reporting.

Secondly, five of the eighteen trials mentioned the presence or absence of adverse effects. The three trials only mentioned that there were no adverse effects in their study. The other two trials reported the presence of adverse effects. One trial [22] reported slight cough (*n* = 1) in the treatment group. Another trial [18] indicated that the treatment group presented with hypoglycemia (*n* = 1) and gastrointestinal tract reaction (*n* = 1) and that the control group presented with hypoglycemia (*n* = 2) and gastrointestinal tract reaction (*n* = 2). The safety of LDP needs to be rigorously monitored in future clinical trials. Therefore, conclusions about the safety of LDP cannot be made from this study due to the limited and inadequate evidence in included trials. Large-scale clinical trials with long-term follow-up appear warranted.

Thirdly, none of the eighteen included trials mentioned health related quality of life, economic index, or compliance with treatments. A recent investigation of 20 public hospitals and integrative medical hospitals in Beijing demonstrated that $4 million can be saved in medical expenses if prescriptions of Chinese herbal medicine were increased by 1% [33]. In view of this finding, we should pay attention to the health economics indices of LDP treatment.

## 5. Conclusions

The results suggest that Liuwei Dihuang Pills added to other routine treatment have a therapeutic potential for people with diabetic nephropathy. However, due to the poor quality of included studies, the reported effectiveness and safety of Liuwei Dihuang Pills for diabetic nephropathy cannot be taken as confirmative conclusion. More rigorous RCTs will be needed to present a high level of evidence for the effectiveness of Liuwei Dihuang Pills in treating diabetic nephropathy.

## Figures and Tables

**Table 1:** Characteristics and methodological quality of included studies.

Reference (year)	Study design	Participants T/C	Intervention (herbs included)	Control	Outcome measure	Treatment duration (days)
Cao (2013) [18]	RCT	36/36	LDP treatment (6 g, bid) plus acarbose (100 mg, tid)	Acarbose (100 mg, tid)	24 h UTP; FBG; PBG; UmAlb	60

Lou (2014) [32]	RCT	60/60	LDP treatment (8 pills, tid) plus telmisartan (80 mg, qd) plus routine treatment	Telmisartan (80 mg, qd) plus routine treatment	24 h UTP	360

Li and Zhao (2011) [26]	RCT	30/30	LDP treatment (8 pills, tid) plus routine treatment	Routine treatment	UAER	90

He et al. (2009) [20]	RCT	17/18	LDP treatment (8 pills, tid) plus Benazepril Hydrochloride (10 mg, qd)	Benazepril Hydrochloride (10 mg, qd)	24 h UTP	90

Song et al. (2004) [28]	RCT	41/31	LDP treatment (9 g, tid) plus routine treatment	Routine treatment	FBG, PBG, UAER	90

Chen and Ling (2004) [15]	RCT	38/30	LDP treatment (6 g, tid) plus captopril (12.5 mg, tid) plus routine treatment	Captopril (12.5 mg, tid) plus routine treatment	UAER	90

Liu (2012) [21]	RCT	30/30	LDP treatment (9 g, bid) plus routine treatment	Routine treatment	BUN, Scr	60

Kong and Zeng (2014) [17]	RCT	25/25	LDP treatment (8 pills, tid) plus routine treatment	Routine treatment	FBG, PBG, HbA1c	360

Chen et al. (2009) [23]	RCT	34/34	LDP treatment (10 g, bid) plus routine treatment	Routine treatment	UAER, FBG, BUN, Scr	60

Wang (2015) [24]	RCT	50/50	LDP treatment (9 g, bid) plus Losartan Potassium (50 mg, qd)	Losartan Potassium tablets (50 mg, qd)	Scr, BUN, UmAlb, UAER	56

Wang et al. (2013) [16]	RCT	33/30	LDP treatment (6 g, bid) plus valsartan dispersible (80 mg, qd) plus routine treatment	Valsartan dispersible (80 mg, qd) plus routine treatment	UAER, UmAlb	28

Yin et al. (2010) [29]	RCT	17/17	LDP treatment (6 g, bid) plus routine treatment	Routine treatment	FBG, UmAlb	56

Li (2013) [22]	RCT	37/38	LDP treatment (8 pills, tid) plus enalapril (5 mg, bid)	Enalapril (5 mg, bid)	FBG, UmAlb, HbA1c	42

Zhong et al. (2010) [30]	RCT	21/21	LDP treatment (6 g, bid) plus routine treatment	Routine treatment	Scr	21

Zhao (2010) [25]	RCT	30/30	LDP treatment (8 pills, tid) plus routine treatment	Routine treatment	24 h UTP	90

Ma (2015) [31]	RCT	74/74	LDP treatment (8 pills, tid) plus Losartan Potassium (50 mg, qd)	Losartan Potassium tablets (50 mg, qd)	UAER, Scr, BUN	180

Deng et al. (2006) [19]	RCT	22/22	LDP treatment (10 g, tid) plus Perindopril (2 mg, qd) plus routine treatment	Perindopril (2 mg, qd) plus routine treatment	UmAlb	56

Lv and Wang (2006) [27]	RCT	45/41	LDP treatment (8 pills, tid) plus routine treatment	Routine treatment	24 h UTP, UAER, Scr	112

**Table 2:** Quality assessment of included randomized controlled trials.

Included trials	Random sequence generation	Allocation concealment	Blinding of participants and personnel	Blinding of outcome assessment	Incomplete outcome data	Selective reporting	Other sources of bias
Cao (2013) [18]	Unclear	Unclear	Unclear	Unclear	No	No	Unclear

Lou (2014) [32]	Table of random numbers	Unclear	Unclear	Unclear	No	No	Unclear

Li and Zhao (2011) [26]	Unclear	Unclear	Unclear	Unclear	No	No	Unclear

He et al. (2009) [20]	Unclear	Unclear	Unclear	Unclear	No	No	Unclear

Song et al. (2004) [28]	Table of random numbers	Unclear	Unclear	Unclear	No	No	Unclear

Chen and Ling (2004) [15]	Unclear	Unclear	Unclear	Unclear	No	No	Unclear

Liu (2012) [21]	Unclear	Unclear	Unclear	Unclear	No	No	Unclear

Kong and Zeng (2014) [17]	Order of hospital registration	Unclear	Unclear	Unclear	No	No	Unclear

Chen et al. (2009) [23]	Order of hospital registration	Unclear	Single blinding	Unclear	No	No	Unclear

Wang (2015) [24]	Drawing lots	Unclear	Unclear	Unclear	No	No	Unclear

Wang et al. (2013) [16]	Coin tossing	Unclear	Unclear	Unclear	No	No	Unclear

Yin et al. (2010) [29]	Unclear	Unclear	Unclear	Unclear	No	No	Unclear

Li (2013) [22]	Table of random numbers	Unclear	Unclear	Unclear	No	No	Unclear

Zhong et al. (2010) [30]	Table of random numbers	Unclear	Unclear	Unclear	No	No	Unclear

Zhao (2010) [25]	Unclear	Unclear	Unclear	Unclear	No	No	Unclear

Ma (2015) [31]	Unclear	Unclear	Unclear	Unclear	No	No	Unclear

Deng et al. (2006) [19]	Unclear	Unclear	Unclear	Unclear	No	No	Unclear

Lv and Wang (2006) [27]	Unclear	Unclear	Unclear	Unclear	No	No	Unclear

**Table 3:** Analysis of the score of FBG.

Trials		MD (95% CI)	*P* value
LDP treatment plus Western drugs versus Western drugs			
LDP treatment plus acarbose versus acarbose	1	−1.05 [−1.78, −0.32]	0.005
LDP treatment plus enalapril versus enalapril	1	−0.39 [−0.53, −0.25]	<0.00001
LDP treatment plus routine treatment versus routine treatment	1	−0.66 [−1.27, −0.05]	0.03
LDP treatment plus routine treatment versus routine treatment	1	−0.15 [−1.48, 1.18]	0.83
LDP treatment plus routine treatment versus routine treatment	1	−0.20 [−0.41, 0.01]	0.06
LDP treatment plus routine treatment versus routine treatment	1	−0.31 [−0.76, 0.14]	0.17
Meta-analysis	6	−0.36 [−0.46, −0.25]	<0.00001

**Table 4:** Analysis of the score of PBG.

Trials		MD (95% CI)	*P* value
LDP treatment plus Western drugs versus Western drugs			
LDP treatment plus acarbose versus acarbose	1	−1.49 [−2.93, −0.05]	0.04
LDP treatment plus routine treatment versus routine treatment	1	−1.49 [−2.93, −0.05]	0.68
LDP treatment plus routine treatment versus routine treatment	1	−1.10 [−1.35, −0.85]	<0.00001
Meta-analysis	3	−1.10 [−1.35, −0.85]	<0.00001

**Table 5:** Analysis of the score of HbA1c.

Trials		MD (95% CI)	*P* value
LDP plus antihypertensive drugs versus antihypertensive drugs			
LDP treatment plus enalapril versus enalapril	1	−0.13 [−0.50, 0.24]	0.49
LDP treatment plus routine treatment versus routine treatment	1	−0.20 [−1.15, 0.75]	0.68
Meta-analysis	2	−0.14 [−0.49, 0.21]	0.43

**Table 6:** Analysis of the score of BUN.

Trials		MD (95% CI)	*P* value
LDP plus routine treatment drugs versus routine treatment drugs			
LDP treatment plus routine treatment versus routine treatment	1	−1.54 [−3.07, −0.01]	0.05
LDP treatment plus routine treatment versus routine treatment	1	−0.57 [−1.98, 0.84]	0.43
LDP treatment plus Losartan Potassium plus Losartan Potassium	1	−0.88 [−1.15, −0.61]	<0.00001
LDP treatment plus Losartan Potassium plus Losartan Potassium	1	−0.08 [−0.51, 0.35]	0.71
Meta-analysis	4	−0.63 [−1.24, −0.02]	0.04

**Table 7:** Analysis of the score of serum creatinine.

Trials		MD (95% CI)	*P* value
LDP plus conventional drugs versus conventional drugs			
LDP treatment plus routine treatment versus routine treatment	1	−0.85 [−1.35, −0.36]	0.0008
LDP treatment plus routine treatment versus routine treatment	1	−0.21 [−0.72, 0.30]	0.41
LDP treatment plus routine treatment versus routine treatment	1	−0.65 [−1.29, −0.01]	0.05
LDP treatment plus routine treatment versus routine treatment	1	−0.71 [−1.34, −0.08]	0.03
Meta-analysis	4	−0.59 [−0.87, −0.32]	<0.0001
LDP treatment plus Losartan Potassium versus Losartan Potassium	1	−1.81 [−2.19, −1.42]	<0.00001
LDP treatment plus Losartan Potassium versus Losartan Potassium	1	−0.46 [−0.86, −0.07]	0.02
Meta-analysis	2	−1.14 [−2.45, 0.18]	0.09

**Table 8:** Analysis of the score of 24 h UTP.

Trials		SMD (95% CI)	*P* value
LDP plus Western drugs versus Western drugs			
LDP treatment plus acarbose versus acarbose	1	−0.48 [−0.95, −0.01]	0.04
LDP plus Benazepril Hydrochloride versus Benazepril Hydrochloride	1	−0.80 [−1.49, −0.11]	0.02
LDP plus routine treatment versus routine treatment	1	−0.89 [−1.55, −0.24]	0.007
LDP plus routine treatment versus routine treatment	1	−0.67 [−1.19, −0.15]	0.01
Meta-analysis	4	−0.67 [−0.95, −0.38]	<0.00001
LDP plus telmisartan plus routine treatment versus telmisartan plus routine treatment	1	−3.48 [−4.05, −2.90]	<0.00001
Meta-analysis	1	−3.48 [−4.05, −2.90]	<0.00001

**Table 9:** Analysis of the score of UmAlb.

Trials		SMD (95% CI)	*P* value
LDP plus conventional drugs versus conventional drugs			
LDP plus captopril plus routine treatment versus captopril plus routine treatment	1	−1.52 [−2.07, −0.98]	<0.00001
LDP plus Losartan Potassium versus Losartan Potassium	1	−1.25 [−1.68, −0.82]	<0.00001
LDP plus routine treatment versus routine treatment	1	−1.47 [−2.24, −0.70]	0.0002
Meta-analysis	3	−1.37 [−1.68, −1.06]	<0.00001
LDP plus Perindopril plus routine treatment versus Perindopril plus routine treatment	1	−4.70 [−5.89, −3.52]	<0.00001
Meta-analysis	1	−4.70 [−5.89, −3.52]	<0.00001
LDP plus enalapril versus enalapril	1	−0.27 [−0.73, 0.18]	0.24
Meta-analysis	1	−0.27 [−0.73, 0.18]	0.24

**Table 10:** Analysis of the score of UAER.

Trials		MD (95% CI)	*P* value
LDP plus Western drugs versus Western drugs			
LDP plus routine treatment versus routine treatment	1	−51.30 [−64.13, −38.47]	<0.00001
LDP plus routine treatment versus routine treatment	1	−34.40 [−44.40, −24.40]	<0.00001
LDP plus routine treatment versus routine treatment	1	−24.88 [−37.94, −11.82]	0.0002
LDP plus routine treatment versus routine treatment	1	−44.40 [−46.59, −42.21]	<0.00001
Meta-analysis	4	−43.65 [−45.73, −41.58]	<0.00001
LDP plus Losartan Potassium plus Losartan Potassium	1	−27.76 [−30.70, −24.82]	<0.00001
LDP plus Losartan Potassium plus Losartan Potassium	1	−24.81 [−31.67, −17.95]	<0.00001
Meta-analysis	2	−27.30 [−30.01, −24.60]	<0.00001
LDP plus captopril plus routine treatment versus captopril plus routine treatment	1	−42.84 [−56.40, −29.28]	<0.00001
LDP plus valsartan dispersible plus routine treatment versus valsartan dispersible plus routine treatment	1	−3.51 [−5.81, −1.21]	0.003
Meta-analysis	2	−4.61 [−6.88, −2.34]	<0.0001

## References

[1] Scully T. (2012). Diabetes in numbers. *Nature*.

[2] Glandt M., Bloomgarden Z. T. (2011). Hypertension in diabetes: treatment considerations. *The Journal of Clinical Hypertension*.

[3] Kolasinska-Malkowska K., Filipiak K. J., Gwizdala A., Tykarski A. (2008). Current possibilities of ACE inhibitor and ARB combination in arterial hypertension and its complications. *Expert Review of Cardiovascular Therapy*.

[4] Wang J., Xiong X. J. (2012). Current situation and perspectives of clinical study in integrative medicine in China. *Evidence-Based Complementary and Alternative Medicine*.

[5] Lim A. K. H. (2014). Diabetic nephropathy—complications and treatment. *International Journal of Nephrology and Renovascular Disease*.

[6] Heerspink H. J. L., de Zeeuw D. (2011). The kidney in type 2 diabetes therapy. *Review of Diabetic Studies*.

[7] Wang J., Wang P. Q., Xiong X. J. (2012). Current situation and re-understanding of syndrome and formula syndrome in Chinese medicine. *Internal Medicine*.

[8] Shi X., Lu X. G., Zhan L. B. (2011). The effects of the Chinese medicine ZiBu PiYin recipe on the hippocampus in a rat model of diabetes-associated cognitive decline: a proteomic analysis. *Diabetologia*.

[9] Zhao H.-L., Sui Y., Qiao C.-F. (2012). Sustained antidiabetic effects of a berberine-containing Chinese herbal medicine through regulation of hepatic gene expression. *Diabetes*.

[10] Wen X. Y., Zeng Y. L., Liu L. F. (2012). Zhenqing recipe alleviates diabetic nephropathy in experimental type 2 diabetic rats through suppression of SREBP-1c. *Journal of Ethnopharmacology*.

[11] Tu X., Ye X. F., Xie C. G., Chen J., Wang F., Zhong S. (2015). Combination therapy with Chinese medicine and ACEI/ARB for the management of diabetic nephropathy: the promise in research fragments. *Current Vascular Pharmacology*.

[12] Wang J., Yao K. W., Yang X. C. (2012). Chinese patent medicine Liu Wei Di Huang Wan combined with antihypertensive drugs, a new integrative medicine therapy, for the treatment of essential hypertension: a systematic review of randomized controlled trials. *Evidence-Based Complementary and Alternative Medicine*.

[13] Higgins J. P. T., Green S. (2009). *Cochrane Handbook for Systematic Reviews of Interventions,Version 5.1.0*.

[14] Higgins J. P. T., Green S. (2011). *Corchrane Reviewers' Handbook 5.1.0*.

[15] Chen J. L., Ling F. M. (2004). Effect of liuwei dihuang pill on urinary protein in early diabetic nepbropathy. *Journal of New Chinese Medicine*.

[16] Wang Y. J., Chen F. L., Li X., Xiao H. D. (2013). Liuwei dihuang pill combined valsartan treatment of diabetic nephropathy random parallel control study. *Journal of Practical Traditional Chinese Internal Medicine*.

[17] Kong D. D., Zeng Z. F. (2014). Clinical observation of treatment of Liuwei Dihuang pill as an adjuvant therapy to diabetic nepbropathy. *Ningxia Medical Journal*.

[18] Cao Z. H. (2013). Clinical observation of acarbose combined with Liuwei Dihuang Pill in treatment of early diabetic nephropathy. *Drugs & Clinic*.

[19] Deng X. M., Tang A. H., Zhou W. H. (2006). Clinical curative effcet observation of treatment of early diabetic nephropathy combining perindopril with liuwei dihuang pill. *Journal of Sichuan of Traditional Chinese Medicine*.

[20] He J. C., Dong Y., Dong H. J. (2009). Clinical investigation of effect of Liuwei Dihuang pill on patient's renal function sufferring from early diabetic nephropathy combined with hyperhomocysteinemia. *Lishizhen Medicine and Materia Medica Research*.

[21] Liu C. J. (2012). Curative effect observation of treatment of liuwei dihuang pill as an adjuvant therapy to type-2 diabetic nepbropathy. *Journal of Practical Traditional Chinese Medicine*.

[22] Li Z. C. (2013). Curative effect observation of treating senile diabetic nephropathy by Liuwei Dihuang pill and enalapril. *Asia-Pacific Traditional Medicine*.

[23] Chen B., Liu X. P., Wu X. F. (2009). Clinical research of treatment of Liuwei Dihuang pill as an adjuvant therapy to diabetic nepbropathy. *China Practical Medicine*.

[24] Wang J. X. (2015). Clinical research on treating early diabetic nephropathy with liuwei dihuang pill and losartan potassium. *Drugs & Clinic*.

[25] Zhao L. J. (2010). A clinical study on treating early type 2 diabetic nepbropathy with Liuwei Dihuang pill. *Clinical Journal of Chinese Medicine*.

[26] Li X. X., Zhao R. M. (2011). Curative effect observation on treating early diabetic nepbropathy with liuwei dihuang pill. *Chinese Medical Innovations*.

[27] Lv Y., Wang Y. P. (2006). Effect of Liuwei Dihuang pill on kidney function of patients with diabetic nephropathy. *Modernization of Traditional Chinese Medicine and Materia Materia-World Science and Technology*.

[28] Song X.-Y., Chen Q., Qi X.-Y. (2004). Effect of liuwei dihuang pill on erythrocyte aldose reductase activity in early diabetic nephropathy patients. *Chinese Journal of Integrated Traditional and Western Medicine*.

[29] Yin S. L., Wang X. H., Zhao C. Y. (2010). Effect of Liuwei Dihuang pill combined with XueZhiKang capsules to early diabetic nephropathy. *Chinese Journal of Misdiagnostics*.

[30] Zhong R. K., Qiu D. L., Duan Y. F., Wang C. H. (2010). Clinical observation on treating diabetic nephropathy with Liuwei Dihuang pill. *Guangdong Medical Journal*.

[31] Ma Y. P. (2015). A clinical study on treating early type 2 diabetic nepbropathy with losartan potassium and liuwei dihuang pill. *Modern Journal of Integrated Traditional Chinese and Western Medicine*.

[32] Lou J. M. (2014). Effect of telmisartan combined with Liuwei Dihuang pill on pulse pressure and proteinuria of patients sufferring from hypertensive and diabetic nephropathy. *Chinese Journal of Clinical Rational Drug Use*.

[33] New Beijing Newspaper Beijing public hospital first put forward principal of allocating funds depending on the service quality of characteristics. http://health.people.com.cn/GB/10063135.html.

